# 

**Published:** 1912-06-08

**Authors:** 


### Books Received.

H. K. Lewis: " The Care of Infants and Young Children in Health," by M. M. Burgess. Second edition; " For and Against Experiments on Animals," by Stephen Paget, F.R.C.S.

W. B. Saunders and Co.: " Modern Methods in Nursing," by Sanders.

J. Wright and Sons: " Differential Diagnosis of Main Symptoms," by Herbert French.

Constable and Co.: " Technique of the Teat and Capillary Glass Tube," by Sir A. E. Wright, M.D.

Simpkin, Marshall, Hamilton, Kent and Co.: " Feeding and Care of Infants and Children," by Nurse Hughes.

The Scientific Press, Ltd.: " Insurance Against Consumption," by C. H. Garland.

University Tutorial Press: " Text-Book of Hygiene for Teachers," by R. A. Lyster, M.D.

Williams and Norgate: " Principles of Physiology," by Professor J. G. McKendrick.

Baillière, Tindall and Cox: " Index of Practical Nursing," by J. Basil Cook, M.D., D.P.H.

Methuen and Co.: " Handbook of Nursing," by M. N. Oxford.

J. D. Riedel Co.: " Riedel's Berichte Mentor."

### OUR BUREAU OF INFORMATION.

### Interchange of Charitable Help.

The need for a kind of clearing-house among institutions for various kinds of patients is continually making itself felt. In all homes and hospitals cases are apt to occur which can be dealt with satisfactorily, but in a different sort of institution if only it could be found. Matrons often try to keep in touch with charities which can be depended on to help them through with difficult cases, and there is a considerable amount of give and take among all social workers. "Help me in my difficulty and I will be ready to help you in yours." This is the basis on which many well-finished cures get accomplished. The general hospital is embarrassed by some patient who proves to require a prolonged period of nursing and good food rather than treatment in the wards. On the other hand the home to which the sufferer is ultimately despatched may be speedily reduced to difficulties by some patient suddenly showing symptoms which urgently require skilled medical or surgical treatment, and the principle of "one good turn deserves another" is immediately put in practice by the hospital giving precedence to the case recommended by its coadjutor. But many institutions are strangely isolated and out of the stream as it were of organised charity. They find they must deal with their problems single-handed or refuse help altogether under circumstances which move their compassion.

The Hospital Bureau of Information is well adapted to facilitate a spirit of mutual co-operation among agencies which exist for the friendless and the suffering. Week by week we publish particulars of cases which call for aid but which baffle even trained workers. Often these cases are reported to us by those who are engaged in branches of hospital work, perplexed to know how to deal with patients ineligible for treatment in their own institution. Will managers of institutions help to solve these difficulties and offer timely aid, whenever possible, relying on the good services of the Bureau to make known their wants when problems of a different character assail them in their turn?

### Rules for Correspondents.

1. Every letter, on whatever subject, must be accompanied by the coupon to be cut from the back cover (inside page) of The Hospital, current issue, and must contain the name and address of the correspondent with pseudonym for publication if desired.
2. Replies by post cannot be given, save under exceptional circumstances, at the Editor's discretion.
3. Letters from Approved Homes in reply to special needs published in the Bureau should state terms and full particulars, and be sent prepaid under cover to the Editor of the Bureau with coupon and name enclosed for identification.
4. Proprietors of homes which have not yet been entered on the List of Approved Homes, but have spare accommodation likely to suit special needs, are invited to write for an application form for registration. The fee for registration is 5s.
5. Special needs of every description relating to those who are sick or in difficulties are made known in these columns free of charge, but applications relating to business or employment from correspondents in an independent position must be accompanied by a postal order for 5s.
6. All communications to be addressed to The Editor of The Hospital Bureau of Information, 28 Southampton Street, Strand, London, W.C.

### Needs and Helpers.

### Institution for Youth with Cervical Disease.

"G. H. H.," Cardiff.—Can any of our readers suggest an institution suitable for youth of twenty with cervical disease and sinuses in the neck? General hospitals will not receive such cases, but it is one needing medical and nursing treatment. Five shillings weekly can be paid.—We recommend you to try the Royal Sea Bathing Infirmary, Margate, which is intended for similar cases. The terms are rather higher than you name, but not more than 8s. a week if a governor's letter can be obtained.

### Training for Boy without Arms.

"Incurable."—The case of which you send particulars should command every reader's sympathy. We would ask, therefore, if anyone can give a practical suggestion as to suitable training for a lad, aged thirteen, who has just had both arms amputated at the shoulders, as the result of an accident. The parents are of the poorer working-class, so that no payment could be made, but the boy is bright, lively, and promising. It is earnestly desired to bring him up to be a—comparatively—useful member of society instead of allowing him to drift into a begging cripple. We would suggest that application be made to Lord Mayor Treloar's Cripples' Home and College, Alton, Hants, where deformed children as well as cripples from fourteen to seventeen are received and trained.

### Homes at Moderate Terms.

### For Maternity Case.

Dr. R. (106a).—Wanted, a place within two or three miles of Earl's Court, London, where a confinement case could be taken on very moderate terms. The patient is a French lady with weak heart. She has only small means, but is most highly respectable. We shall be happy to forward prepaid replies from Approved Homes sent under cover to The Bureau, as above.

### Hospital Appliances.

### Apparatus for Making Beef-tea.

"C. N. V."—The apparatus for making beef-tea is in use at the Manchester Royal Infirmary. It was described in a recent number of The Hospital, and is supplied by Slater and Co. The special feature is the hot-water jacket, which is kept boiling by steam, while the beef-tea in the infuser is kept always simmering.

### Potato Peeler for Hospital Without Power.

"Rugby."—A handy machine for turning out 5 lb. a minute by hand-power can be obtained as low as seven guineas. They can be had either with or without necessity for water and drain connection, and are not difficult to manipulate. They stand 3 feet 6 inches high and occupy 18 inches floor space. No properly constructed vegetable peeler is responsible for as much waste of food as unskilled labour, if unsupervised.

### Insurance Queries.

### Nurses and the Insurance Act.

Nurse Sears.—Write to Louis Dick, Esq., Secretary of the Royal National Pension Fund for Nurses, 15 Buckingham Street, Strand, W.C., for particulars of the benefit society now being started for all nurses whether members of the Pension Fund or not. A full account of the Insurance Act as it affects nurses will be found in The Hospital for May 25 and June 1, and we recommend you to study these articles carefully.

### Appointments.

Mr. Donald W. Roy, M.B., F.R.C.S., has been appointed registrar to the Samaritan Free Hospital for Women, Marylebone, and also at the Lying-in Hospital, York Road.

Dr. Frank Edward Tylecote, M.D., D.P.H. (Vict.), M.R.C.P. (Lond.), has been appointed honorary physician to the Salford Royal Hospital in place of the late Professor J. Dixon Mann, M.D., F.R.C.P.

Dr. Edward Burnet, M.D., has been appointed medical officer of health for Tunbridge Wells. Dr. Burnet, who recently resigned the post of medical officer for Leamington, and was on the "short list" for the post of deputy medical officer of health to the London County Council, is an M.B. of Edinburgh.

Dr. Adam Niven Robertson, M.B., has been appointed assistant medical officer for Herefordshire. Holding the M.B. of Edinburgh and the D.P.H. of Cambridge, Dr. Robertson studied at King's College, and his previous appointments include that of house surgeon to the Free Eye Hospital, Nottingham. He has also been R.M.O. at a sanatorium near Edinburgh and at Wrexham General Hospital, North Wales.

### Gifts and Bequests.

Mr. John Gilders, of St. Osyth, has left £200 to the Clacton Cottage Hospital.

Mrs. Elizabeth Tyzack, of Sheffield, has left £200 to the Jessop Hospital for Women, Sheffield.

The Royal Dental Hospital, Leicester Square, has received a donation of £100 from R. Nivison, Esq.

Mr. Daniel Charles Stiebel, merchant, who left £462,877, has bequeathed £500 to the London Hospital.

Prince Alexander of Teck has received £656 for the Middlesex Hospital as the result of the table decorating display recently held at the Ideal Home Exhibition.

Mr. Edward Broadlake Dingley, of Sherborne, Dorset, draper, has left £200 each to the Children's Home, Bonner Road, London, and the Yeatman Hospital, Sherborne.

The Chelsea Hospital for Women has received from the Misses Wheeler three cheques of £100 each towards the Rebuilding Fund; also £10 10s. from the Skinners' Company and the annual subscription of £5 5s. from the Salters' Company.

Lord Donoughmore, the Treasurer of the London Homœopathic Hospital, Great Ormond Street, W.C., has received £500 from Edwin Tate, Esq., for the building of the home for the nurses of the hospital now in occupation. Donations to complete the £10,000 are still required.

Mrs. Elizabeth Greaves, of Nottingham, has bequeathed the residue of her estate, about £40,000, in equal shares to the General Hospital, Nottingham, the Dispensary, Nottingham, the Nottingham and Midland Eye Infirmary, the Samaritan Hospital for Women, Nottingham, the Nottingham and Nottinghamshire Convalescent Homes, and certain other institutions.

The Secretary of the West Bromwich District Hospital has received a cheque for £1,500 from the executors of the late J. T. Johnson, in lieu, and in satisfaction, of the legacy of £1,000 left to the hospital under the will of the above-named testator, on the understanding and condition that such sum shall be utilised for the purpose of endowing a bed in the institution to be called "The John Thomas Johnson of West Bromwich" bed.

### Buildings Contemplated.

Ballymena.—The Guardians have under consideration a scheme for an hospital on the northern side of the workhouse.

Billericay.—The Rural District Council have approved the plans for an isolation hospital, and application is to be made to the Local Government Board for sanction to a loan of £6,213 to cover the necessary outlay.

Bishop Auckland.—The Local Government Board have inquired into the application of the Auckland, Shildon, and Willingdon Joint Hospital Board for sanction to a loan of £6,600 for extending the infectious diseases hospital at Midall Crest and Helmington Row. Mr. R. B. Thompson, Bishop Auckland, prepared the plans.

Blaina.—The Blaina and District Cottage Hospital is to be considerably enlarged from plans by Mr. E. A. Johnson, of Abergavenny.

Bristol.—The Guardians have accepted tenders amounting to £44,776 in connection with the proposed infirmary.

Bromley.—A tender of £13,999 by Mr. J. S. Woodhams, of Bromley, has been accepted for workhouse additions.

Cheltenham.—Mr. T. Malvern, Cheltenham, is architect for children's sick and isolation wards at the workhouse. Tenders are to be submitted before June 5, and particulars may be had from the architect.

Fiume.—The *Zentral-Anzeiger* (Vienna) states that the Fiume municipal authorities have decided to spend £220,800 on an infirmary and £54,100 on an infectious diseases hospital.

Great Yarmouth.—Tenders are invited for a new block at the Guardians' Infirmary on the female side. Particulars may be had from Mr. A. S. Hewitt, A.R.I.B.A., Bank Chambers, Great Yarmouth.

Hemsworth.—A new workhouse is mooted at Hemsworth, and the Guardians have instructed their surveyor to prepare plans for the required buildings.

Huddersfield.—Receiving and short-period wards (lunatic) are projected at the Crosland Moor Workhouse. Messrs. J. Kirk and Son, architects, Huddersfield, will give particulars on application.

Kingseat.—The Asylums Committee of the Aberdeen District Lunacy Board is considering an extension of the Kingseat Asylum.

Lambourn.—The Reading Town Council will apply to the Local Government Board for sanction to a loan for purchasing a site and erecting a children's hospital or sanatorium.

Oswestry.—Application is being made to the Local Government Board for authority to utilise land in Shrewsbury Road as a site for an isolation hospital.

Reading.—Messrs. Chas. Smith and Son designed the new buildings at the Royal Berkshire Hospital which have just been formally opened. The cost was £22,000.

Rochford.—A remodelling of the Rochford Workhouse is projected. Particulars may be had on deposit of one guinea from Mr. W. J. Wood, architect, Southend-on-Sea. References and guarantees are required.

Rugby.—It is proposed to apply for sanction to a loan of £1,500 for the building and equipment of a laundry for the Guardians. A site has been purchased for a girls' home.

Scarborough.—Plans for the construction of a phthisis ward at the Dean Road Workhouse may be inspected at the office of the architect, Mr. J. A. Iveson, 16 Dean Road, Scarborough. Tenders are invited for the work.

Sheffield.—June 3 is the date by which designs for a cripples' home have to be sent in by local architects. The assessors are Messrs. Gibbs and Edwards, FF.R.I.B.A.

Somersetshire.—The Bridgwater Rural District Council is considering a proposal to establish four hospitals in different centres to serve the whole county in case of a smallpox epidemic.

Springfield.—Plans by Messrs. Gillespie and Scott, of St. Andrews, have been approved by the Fife and Kinross Lunacy Board for remodelling the district asylum at a cost of £22,000. The work will proceed immediately the General (Scottish) Board of Lunacy gives its sanction.

Stafford.—At a cost of £5,800 it is proposed to erect a nurses' block at the Stafford Asylum. The County Lunacy Committee have approved the plans and figures.

Wallingford.—Alterations and additions are proposed at the Wallingford and Crowmarsh Isolation Hospital. Tenders are invited and particulars may be obtained from Mr. H. S. Morris, of Broadway Buildings, Reading.

Ware.—Mr. James Farley, engineer, Hertford, has prepared a scheme for heating and hot-water supply at the workhouse. The Local Government Board has approved the plans, and the Guardians will presently invite tenders for the work.

### The Week's Novelties.

### PETOL.

This product is an interesting derivative from peat and has already obtained a large amount of favour. Petol in the form of a salve, a soap, or an ointment has been used for many years and has withstood the encroachments of rival preparations in a manner creditable to its excellent intrinsic properties. Petol is a natural antiseptic oil derived from vegetable sources, smelling pleasantly of peat. Its action on the skin makes it a valuable emollient and very soothing antiseptic. The soap and the salve are prepared with non-irritating bases, and practical experience has shown that these are preparations which do undoubtedly exert a favourable influence on the skin. They are especially recommended for insect bites, sun scorch, simple burns, and certain forms of dermatitis. The makers, Petol Ltd., 17 Hart Street, Bloomsbury, have also brought out a very successful shaving soap which is decidedly pleasant and soothing to use.

